# Alternative Options to Glyphosate for Control of Large *Echinochloa colona* and *Chloris virgata* Plants in Cropping Fallows

**DOI:** 10.3390/plants8080245

**Published:** 2019-07-24

**Authors:** Bill Davidson, Tony Cook, Bhagirath S. Chauhan

**Affiliations:** 1 Department of Primary Industries (NSW DPI), Tamworth Agriculture Institute, Tamworth 2340, New South Wales, Australia; 2 Queensland Alliance for Agriculture and Food Innovation (QAAFI), The University of Queensland, Gatton 4343, Australia

**Keywords:** Australia, herbicide resistance, tank mixtures, sequential applications, *echinochloa colona*, *chloris virgata*

## Abstract

The over-reliance on the herbicide glyphosate for knockdown weed control in fallows under minimum and zero-till cropping systems has led to an increase in populations of glyphosate-resistant weeds. *Echinochloa colona* and *Chloris virgata* are two major grass weeds in the cropping regions of northern New South Wales and southern Queensland, Australia, that have become harder to kill due to a steady rise in the occurrence of glyphosate-resistant weed populations. Therefore, to help growers contain these hard to kill fallow weeds, an alternate approach to glyphosate application is needed. With this purpose in mind, a pot study was carried out during the summer seasons of 2015 and 2016 at the Tamworth Agricultural Institute, Tamworth, NSW, Australia, to evaluate the efficacy of tank mixtures and sequential applications of Group H (4-hydroxyphenylpyruvate dioxygenase (HPPD) inhibitor), Group C (inhibitors of photosynthesis at photosystem II), Group A (ACCase inhibitors) and Group L (photosystem I inhibitor) herbicides on late tillering *E. colona* and *C. virgata* plants. These herbicide groups are a global classification by the Herbicide Resistance Action Committee. Highly effective results were achieved in this study using combinations of Groups H, C, A and L herbicides applied as tank mixtures for controlling large *E. colona* plants. Additionally, sequential applications of Group H, C and A herbicides followed by (fb) paraquat were shown to be very effective on large *E. colona* plants. Late tillering *C. virgata* plants were generally well controlled by tank mixtures, and sequential applications proved to be highly effective on this grass weed as well. Haloxyfop in combination with paraquat as a tank mixture, via sequential application or as a stand-alone treatment, was highly effective for *C. virgata* control; however, using combinations of herbicide groups is the preferred choice when combating herbicide resistant weed populations. There was a clear synergy shown using Group H, Group C and Group A herbicides in combination with the Group L herbicide paraquat in this study for controlling advanced *E. colona* and *C. virgata* plants. These combinations were shown to be successful on plants grown under glasshouse conditions; however; these treatments would need to be tested on plants grown in a field situation to show whether they will be a useful solution for farmers who are trying to control these weeds in fallow.

## 1. Introduction

*Echinochloa colona* (L.) Link and *Chloris virgata* SW. are two major fallow weeds that have proliferated under no-till regimes in the crop growing regions of New South Wales (NSW) and Queensland (QLD), Australia. *E. colona* is a major crop weed of the grain growing regions of southern QLD, and northern NSW. It is an annual species that is green to blue-green in colour, smooth in appearance and grows to 300–750 mm high. It can grow upright or prostrate which allows potential secondary roots to develop from lower nodes. Its flower spikelets can be green or tinged with purple, 2.5–3.5 mm long and awnless [1]. It has no ligule and often has purple stripes on leaves and sheaths [2]. *E. colona* mainly germinates through the warmer months of spring and summer in subtropical Australia. It takes approximately 21–28 days from germination before *E. colona* starts to flower with seeds maturing at 6–7 weeks [3]. Each plant has the potential to produce 42,000 seeds [2]. The flowers of *E. colona* are hermaphrodite and wind pollinated [4]. The ideal temperature for germination of *E. colona* seeds is between 20 and 34 °C [5]. Maximum germination potential is on the soil surface, and the deeper that seed is buried germination percentage decreases [6]. Seed is spread by agricultural machinery and animals and through natural or irrigated water flows [7]. Humid conditions and no-till or minimum till farming systems seem to favour the germination of *E. colona* [6]. *E. colona* is a problem weed of all crops grown through the late spring and summer season. It readily germinates and grows in summer fallows and can also germinate and set seed in spring during winter crop growth. Glyphosate resistance was first confirmed in 2007 in one population of *E. colona* in northern NSW [8]. The continued nondiscriminate use of glyphosate in fallows has led to an increase in glyphosate-resistant populations. As of January 2018, there are 102 confirmed cases of glyphosate resistance [9]. Being a rapidly growing summer weed, it can be prone to moisture stress. Work by Tanpipat et al. found that as *E. colona* becomes more moisture stressed, glyphosate efficacy decreases [10]. Additionally moisture stressed plants have the effect of increasing rain fastness of herbicides, which further complicates control when trying to spray in the thunderstorm season [8]. *E. colona* is a prolific weed that affects summer fallows and summer grown crops. The use of an integrated approach combined with novel research tactics will help combat glyphosate resistance.

*Chloris virgata* is a probmatic weed in the grain growing areas of QLD. It is also becoming more of an issue in NSW crop growing regions, especially in the north as well as some central and southern areas [11]. *C. virgata* is a summer growing annual grass plant that is tufted and grows up to 1 m tall with stems that are erect or semiprostrate. The semiprostrate stems have the potential for rooting at the lower nodes. Leaves are blue-green in colour, 50 to 250 mm long and 3 to 9 mm wide. The seed heads point upwards and have a feathery white appearance due to the stiff white hairs and awns originating from the seeds [12]. The seedlings are erect and stems have a flattened appearance and are green in colour. A single plant can produce up to 6000 seeds [11], and the seeds are 2–3 mm long, have an arrowhead type shape, they are light and can be carried by both wind and water [13]. Optimum germination is when temperatures are at 20 °C nighttime and 30 °C daytime, although seeds will germinate over a broad range of temperatures [13]. *C. virgata* grows quickly with plants potentially setting seed within a 42 day period [11]. Ideal growth conditions are when daytime temperatures are approximately 30 °C [14]. *C. virgata* poses problems in summer fallows and summer crops such as sorghum, and similar to *E. colona*, it can germinate in spring within winter crops making control more challenging. The first glyphosate-resistant case was reported only recently in 2015, and as of January 2018, there are four confirmed cases [9]. The use of single knockdown herbicides in fallows such as glyphosate has led to glyphosate-resistant populations. To arrest the development of continued resistance, alternate tactics will need to be employed. 

Weed populations that are hard to kill or have become herbicide resistant require more complex tactics for their control. Tank mixtures and sequential applications using herbicides from different modes of action (MOA) groups have been successful in controlling hard to kill and/ or herbicide-resistant weeds. The technique of using one weed control tactic followed by another separate tactic, with the aim of the second tactic killing any weeds that were not controlled by the first tactic, is known as a sequential application [2]. Initially, use of this strategy centered on using a knockdown herbicide as the first application, followed by an unrelated technique such as cultivation for the second application. The evolution of cropping systems has shown a preference for reduced or zero cultivation with the aim of preserving moisture for subsequent crops. Therefore, the sequential application method has evolved into two applications of herbicide using different MOA groups for the first and second application. The length of time separating the two applications will vary according to what weeds are being sprayed, the growth stage of weeds and what herbicide MOA groups are being used [2]. Generally, the second application will be applied between 1 and 14 days after the first application. The sequential application method has the potential to slow down the spread of glyphosate resistance. Modelling work on *Lolium rigidum* Gaud. predicted glyphosate resistance in only 17 of the 1000 simulation runs using the scenario of glyphosate followed by paraquat [15]. *E. colona* plants at varying growth stages can be well controlled using the sequential application technique. Over five experiments, a sequential application of glyphosate followed by paraquat achieved 100% control of *E. colona* plants ranging from early to late tillering in the growth stage [16]. In one of the experiments, control dropped to between 96 and 99.4%, which was likely due to the high density of plants being sprayed which may have impeded proper coverage of plants with herbicide [16]. The sequential application technique also worked well on three growth stages of *L. rigidium*, using glyphosate as the first application and paraquat as the second. In this study, sequential applications were more effective than using glyphosate or paraquat as a single treatment [17]. The sequential application method works well on both grasses and broadleaf weeds. In a previous study, a Group I (global classification by Herbicide Resistance Action Committee) herbicide (disrupters of plant cell growth) followed by an application of paraquat 7 days later on *Conyza bonariensis* (L.) Cronq. plants proved to be an effective combination when compared to single applications of herbicides [18]. Sequential application is an effective tool to control populations of herbicide-resistant weeds and hard to kill and/or glyphosate-tolerant weeds. Additionally, this method can help alleviate selection pressure for resistance in selective in-crop herbicides. Reduced numbers of herbicide-resistant weeds resulting from the effectiveness of using sequential applications contribute to less selection pressure for the selective herbicides that are used in-crop [2].

The use of herbicide mixtures can be an effective part of an integrated approach to dealing with resistant weed populations, provided herbicide combinations come from different MOA groups and are synergistic [19]. Herbicide mixtures also have the potential to reduce the chance of selecting for resistance in herbicide-resistant weeds, if the herbicides used are from different MOA groups [20]. Under a modelling scenario, tank mixtures of herbicides from two or more MOA groups appeared to be more effective at slowing down the onset of resistance than rotating herbicides from different groups, especially when areas of weed populations were 100 hectares or less [21]. A tank mixture of propanil with either anilofos or piperophos applied to propanil-resistant *Echinochloa crus*-*galli* (P.) Beav. proved to be very effective in controlling this weed in southern United States rice crops, with limited damage to the crop [22]. The continued use of single applications of herbicides to control fallow weeds has the potential to expedite the onset of herbicide resistance. The longevity of herbicides such as glyphosate may be prolonged if mixed with herbicides from other MOA groups when applying to fallow weeds such as *Sonchus oleraceus* L. [23]. A tank mixture of paraquat plus imazapic was shown to be very effective for the control of *E. colona* plants in two field experiments conducted on at Pittsworth and Dalby, the Darling Downs in north-eastern Australia [16]. Control levels ranged from 96–99% when assessed using a biomass reduction score (% of untreated plots). 

A tank mixture of the Group H (4-hydroxyphenylpyruvate dioxygenase (HPPD) inhibitor) herbicide tembotrione and Group C (photosystem II inhibitor) herbicide atrazine was synergistic for controlling large *Digitaria sanguinalis* (L.) Scop, *Panicum miliaceum* L., *Chenopodium album* L., and *Abutilon theophrasti* plants in maize crop experiments in Illinois, Oregon, Washington, U.S.A. and Ontario, Canada [24]. This tank mix treatment was superior by 3% to 45% to single applications for weed control in this experiment. Control of propanil-resistant *E. colona* was improved by tank mixing propanil with the Group D (microtubule inhibitors) herbicide pendimethalin in glasshouse and field experiments in Costa Rica [25]. Tank mixtures of pendimethalin and propanil lowered rates of herbicide actives needed to control three-to-four leaf resistant *E. colona* plants. Pendimethalin and propanil tank mixed at rates of 0.23 kg ha^−1^ and 1.08 kg ha^−1^, respectively, exhibited similar levels of control to 2.16 kg ha^−1^ of propanil applied alone [25].

Paraquat is routinely used as the final herbicide in the sequential application technique, predominantly because of its dissimilar MOA to the systemic herbicides used in the first application. Paraquat is widely reported as being effective for this purpose and plays a significant part in this successful tactic for controlling hard to kill and or glyphosate-resistant weeds. A tank mix of systemic herbicides followed by a paraquat-based herbicide as the second application 7 days later, was found to be very effective for controlling varying growth stages of *C. bonariensis* plants [26]. The use of sequential application treatments using paraquat to control glyphosate-resistant *E. colona* proved to be potent as well, showing the usefulness of this tactic on fallow grass weeds [8]. A study on *C. bonariensis* using the sequential application technique using paraquat as the secondary herbicide was very effective for controlling this problematic weed [18]. Glyphosate-resistant *S. oleraceus* can also be successfully controlled in the field using paraquat as the second application [27]. 

The following pot study was carried out to gauge whether tank mixtures and/or sequential applications specifically using Group H (4-hydroxyphenylpyruvate dioxygenase (HPPD) inhibitor), Group C (inhibitors of photosynthesis at photosystem II), Group A (ACCase inhibitors) and Group L (photosystem I inhibitor) herbicides are effective for controlling advanced *E. colona* and *C. virgata* plants. Advanced plants, nearing or in a reproductive stage, provide a more robust test for treatments than smaller plants and more closely resemble the portion of larger weeds farmers would have in their paddocks after harvesting crops. 

## 2. Materials and Methods

### 2.1. Experimental Location and Potting Mix Type

The glasshouse experiments were conducted during summer of 2015 and 2016 at the Tamworth Agricultural Institute, Tamworth (31.0927° S, 150.9320° E), NSW, Australia. The glasshouse was not temperature controlled; however, evaporative cooling did avoid extremes of hot and cold temperatures. The potting mix used for germination of weed seedlings and the duration of the experiment was a premium mix made by Scotts Australia Pty Ltd, Bella Vista, Australia. The potting mix comprised of composted hardwood sawdust and pine bark, cocopeat fibre and nutrients consisting of calcium, ammonium nitrate, gypsum, iron sulfate, potassium sulfate, phosphorus and sulphur. In addition, this potting mix contained water storage crystals and a wetting agent. 

### 2.2. Weed Plant Management 

Seeds of *E. colona* were collected from plants grown in the glasshouse in the autumn of 2014 from a population originally sourced from a property near Bellata (29.9019° S, 149.7884° E), NSW, Australia. The *C. virgata* seeds were collected off plants located in paddocks on the Tamworth Agricultural Institute research farm in 2015. *E. colona* was susceptible to glyphosate but *C. virgata* is not mentioned on the label of glyphosate. After collection, seeds from both species were placed in paper envelopes and stored at room temperature. To commence the experiments, seeds were germinated in shallow plastic trays filled with premium potting mix. When seedlings reached the three-leaf growth stage, they were transplanted as single plants into 8 cm square and 15 cm deep pots. These potted plants were then placed in trays with water so as to eliminate moisture stress, and fertilised with a soluble plant fertiliser every 3 weeks up until plants reached late tillering to inflorescence emergence (Z50–59). The fertiliser comprised of 25% nitrogen, 5% phosphorous, 8.8% potassium, 4.6% sulphur, 0.5% magnesium, 0.18% iron, 0.01% manganese, 0.005% boron, 0.005% copper, 0.004% zinc and 0.001% molybdenum. Once plants reached late tillering to inflorescence emergence (Z50–59), they were moved into trays containing water outside the glasshouse for 14 days (d) before herbicide application. This process was carried out so that plants were exposed to weather conditions similar to paddock plants before herbicide application.

### 2.3. Treatments and Experimental Design

There were four tank mixture application and four sequential application experiments carried out in this study. The treatments were the same for both *E. colona* and *C. virgata*; thus, there was a tank mixture application and a sequential application experiment for each species in each year. This resulted in four experiments per year and eight experiments in total over the two years. The herbicides, mode of action (MOA) herbicide group and herbicide doses are shown in Table 1 and Table 2. The herbicide treatments were applied using a powered backpack sprayer with a 2 m wide four-nozzle boom, delivering 100 L ha^−1^ of spray solution. Nozzles used were flat fan with an orifice size of 02 (ASAE S-572 spray tip classification) and a spray coverage angle of 110 degrees. After herbicide application, pots were taken back into the glasshouse for the duration of the experiment. The tank mixture experiments had 12 treatments, comprising of 11 herbicide treatments and one nontreated, with every treatment replicated six times. The sequential application experiment had 13 treatments, comprising of 12 herbicide treatments and one nontreated, with every treatment replicated six times. The timing of the sequential application treatments was 7 d between the first and second applications. Tank mixture treatments involved mixing of herbicides in a single tank mixture and spraying as one application.

### 2.4. Sampling Techniques and Observations

Herbicide efficacy was evaluated at 42 d after herbicide treatment for each experiment. Plants alive at 42 d after herbicide application had any remaining green plant material extracted and placed into a separate paper bag for drying. The paper bags containing the green plant material were then oven-dried at 80 °C for a minimum of 72 h. At the completion of the drying period, plant material was removed from the paper bag and weighed. Plants that were completely dead at 42 d after treatment were given a value of zero for green biomass.

### 2.5. Statistical Analyses

Experiments were conducted using a completely randomized design with six replications in each year. Experimental data was analysed using a generalised linear modelling program called ASReml (VSN International Ltd, Hemel Hempstead, UK). Comparisons of means were implemented based on the least significant difference test at 0.05 probability. Original values were included in the analysis as transformation did not significantly improve homogeneity of variance. Therefore, original data values are presented. Data are presented separately for each year to better understand variation between the years.

## 3. Results and Discussion

### 3.1. Effect of Tank Mixture Herbicide Treatments on Echinochloa colona

The tank mixtures of isoxaflutole plus paraquat, atrazine plus paraquat, simazine plus paraquat and terbuthylazine plus paraquat were very effective in controlling large *E. colona* plants, with complete control at 42 d after treatment in the 2015 experiment. These treatments were also effective in the 2016 experiment, exhibiting high control levels on large *E. colona* plants (Table 3). Additionally, the haloxyfop plus paraquat tank mixture was very effective and had similar results compared to the other tank mixture treatments over both experiments. The tank mixture treatments gave better (*p* < 0.05) control than the single herbicide treatments with the exception of paraquat. Paraquat as a standalone treatment was very effective over both experiments with similar efficacy levels to the tank mixture treatments. 

The single herbicide treatments of isoxaflutole, atrazine, simazine, terbuthylazine and haloxyfop had lower weed biomass compared to nontreated *E. colona*; however, they were inferior to the tank mix treatments. Furthermore, haloxyfop was similar to the tank mixture treatments in the 2016 experiment with 91% control. Predominantly, the tank mixture treatments were clearly superior to the single herbicide treatments. The addition of paraquat to the single herbicides and combining as a tank mix appears to have had a synergistic effect for controlling large *E. colona* plants. Paraquat as a stand-alone treatment was similar in efficacy to the tank mixture treatments, which may question the practice of using herbicides in combination when paraquat is just as potent. One feasible justification for using a tank mixture over paraquat is that the use of herbicides from different MOA groups combined as a tank mixture will slow down selection to resistance [19,20]. Furthermore, tank mixtures of paraquat with systemic herbicides may have improved performance when compared to paraquat alone. Previous studies on control of glyphosate-resistant *C. canadensis* in a fallow paddock at Leland, Mississippi, USA pointed to tank mixtures being more effective than paraquat as a stand-alone treatment [28]. Tank mixtures of paraquat plus 2,4-D and paraquat plus dicamba resulted in 88 and 89% control levels, respectively, compared to 63% control for the paraquat treatment [28].

### 3.2. Effect of Sequential Application Herbicide Treatments on Echinochloa colona 

All of the sequential treatments were highly effective for controlling large *E. colona* plants over the 2015 and 2016 experiments. Isoxaflutole fb an application of paraquat 7 days later achieved 99% control in 2015 and 100% control in 2016 at 42 d (Table 4). Atrazine fb paraquat, simazine fb paraquat, terbuthylazine fb paraquat, haloxyfop fb paraquat and paraquat fb paraquat all achieved very high levels of control on large *E. colona* at 42 d. Paraquat was the only single product treatment that was effective in controlling *E. colona* with control levels similar to the sequential treatments. The remaining single treatments of isoxaflutole, atrazine, simazine, terbuthylazine and haloxyfop were inferior to the sequential application treatments and the single treatment of paraquat. 

Generally, results were mainly consistent across both experiments with the exception of few treatments which showed a sizeable disparity between the 2015 and 2016 experiments. A possible reason for this disparity in results may be due to differences in humidity when treatments were applied to plants from the separate experiments. If temperatures are similar but humidity is significantly different, especially when spraying in warmer times of the year, then herbicide uptake can be affected [29]. There have been documented cases of this occurring when using the herbicide glufosinate on grass species such as *Hordeum vulgare* L. cv. ‘Samson’ and *Setaria viridis* (L.) Beauv [29]. In a glasshouse study, it was found that the efficacy of glufosinate ammonium applied at 100 g ha^−1^ on *S. viridis* was significantly reduced in conditions with lower humidity, even though temperatures were similar. When this herbicide was applied at 40% relative humidity, the *S. viridis* plants accrued 70% of the dry weight of nontreated plants; however, when the same herbicide at the same rate was applied under 95% relative humidity, plants were totally killed [30]. Differential plant size between the 2015 and 2016 experiments may also influence herbicide efficacy. 

### 3.3. Effect of Tank Mixture Herbicide Treatments on Chloris virgata

The tank mix treatments of isoxaflutole plus paraquat, atrazine plus paraquat, simazine plus paraquat, terbuthylazine plus paraquat and haloxyfop plus paraquat were effective in controlling large *C. virgata* plants across the two experiments (Table 5). 

The tank mix combination of haloxyfop and paraquat was the only treatment to achieve 100% control in both the 2015 and 2016 experiments. The tank mixing of paraquat with single herbicides was shown to be synergistic for controlling large *C. virgata,* plants. Tank mixes were more effective (*p* < 0.05) than the single herbicide treatments, with the exception of the single haloxyfop treatment, which achieved 98% and 99% control, respectively, across the two years. Generally, the tank mixes were superior to paraquat, although in the 2016 experiment it had similar control levels to the tank mix treatment treatments, excluding isoxaflutole plus paraquat and haloxyfop plus paraquat which were more effective. Previous research indicates that single knockdown herbicides such as glyphosate will not effectively control large *C. virgata* plants [12]. In previous work involving on-farm winter fallow experiments in central Queensland, Australia, control of *C. virgata* was improved using a combination of herbicides from different MOA groups rather than single herbicides alone [12]. Although this work was carried out on earlier weed growth stages, it appears as though there is a synergistic effect of mixing certain herbicides for control of *C. virgata* regardless of its size.

### 3.4. Effect of Sequential Application Herbicide Treatments on Chloris virgata

The sequential application treatments of isoxaflutole fb paraquat, atrazine fb paraquat, simazine fb paraquat, terbuthylazine fb paraquat and haloxyfop fb paraquat were very effective in controlling large *C. virgata* plants across the 2015 and 2016 experiments (Table 6). 

Isoxaflutole fb paraquat and haloxyfop fb paraquat achieved 100% control over both years. In the 2016 experiment, atrazine fb paraquat and paraquat fb paraquat also achieved 100% control, with no weed biomass remaining at 42 d. All of the sequential application treatments had similar levels of control. The sequential application treatments were clearly superior to the single herbicide treatments, although paraquat as a stand-alone treatment in the 2016 experiment had similar efficacy to the sequential application treatments with 91% control at 42 d. Paraquat fb paraquat achieved 96% and 100% control, respectively, over the 2015 and 2016 experiments which shows the benefit of using two applications of paraquat 7 days apart over a single application. As suggested previously, although paraquat as a single stand-alone treatment has been moderately effective in controlling *C. virgata* plants in this study, the use of mixtures and or sequential applications is the preferred option. Previous studies under glasshouse and field conditions conducted in Toowoomba, southern Queensland, showed that sequential applications of systemic herbicides followed by paraquat were more effective than single applications [18]. Additional work from central Queensland conducted in cropping fallows showed that single applications of herbicides were inferior to sequential applications for the control of *C. virgata* at a range of growth stages [12].

## 4. Conclusions

Tank mixtures of Group A, C, and H herbicides mixed with the Group L herbicide paraquat were very effective in controlling large *E. colona* plants, showing a synergism between the systemic MOA herbicide groups and Group L. Additionally, paraquat as a stand-alone treatment had high efficacy levels, and haloxyfop was effective in one experiment and not the other, which may have been due to varying humidity levels at time of application, as mentioned previously. Overall, sequential treatments of Group A, C, and H herbicides followed by paraquat 7 d later were very effective in controlling large *E. colona* plants. Results from this study suggest that large *E. colona* plants can be well controlled with tank mixtures of Group A, C, H and L herbicides and sequential applications of Group A, C, and H followed by a Group L herbicide 7 days later, and are generally superior to single herbicide applications. This synergism between the MOA groups provides an important alternative control option to manage large *E. colona* plants in fallow situations. Tank mixture treatments effectively controlled large *C. virgata* plants and were markedly better than the single herbicide treatments with the exception of haloxyfop. Interestingly, haloxyfop as a single treatment achieved excellent control in both experiments. Haloxyfop plus paraquat was the most effective tank mix treatment with 100% control for both experiments at 42 d. The sequential treatments were superior to the single treatments for controlling large *C. virgata* plants; however, haloxyfop on its own was very effective with 96% and 98% control, respectively, over the 2015 and 2016 experiments. There seems to be a clear synergistic effect of combining Group A, C, and H herbicides with the Group L herbicide paraquat, as either tank mixes or sequential applications to control large *E. colona* and *C. virgata* plants. It is quite feasible that these treatments will not be as effective under field conditions, due to plants being more stressed under paddock conditions and consequently some adjustments of treatments may be required, possibly through the addition of other herbicide groups or an increase in rate of active. If, however, these treatments are effective under field conditions, then there is promise to use these herbicide combinations as an alternative to glyphosate to managing these weeds in a fallow scenario. The use of Group H and C herbicides when mixed with paraquat do provide the additional benefit of residual activity in conjunction with post-emergent control. The use of these herbicides comes with inherent risk to subsequent crop safety, and more thorough planning would be required to integrate them into crop management systems.

## Figures and Tables

**Table 1:** Herbicide treatments, mode of action (MOA) herbicide group, and herbicide dose used in tank mixture experiments.

Herbicide Treatment	MOA Groups	Dose (g ai ha^−1^)
Nontreated		
Isoxaflutole	H	75
Isoxaflutole plus paraquat	H plus L	75 plus 500
Atrazine	C	2997
Atrazine plus paraquat	C plus L	2997 plus 500
Simazine	C	1494
Simazine plus paraquat	C plus L	1494 plus 500
Terbuthylazine	C	750
Terbuthylazine plus paraquat	C plus L	750 plus 500
Haloxyfop	A	156
Haloxyfop plus paraquat	A plus L	156 plus 500
Paraquat	L	500

**Table 2:** Herbicide treatments, mode of action (MOA) herbicide group and herbicide dose used in sequential application experiments.

Herbicide Treatment	MOA Groups	Dose (g ai ha^−1^)
Nontreated		
Isoxaflutole	H	75
Isoxaflutole fb paraquat	H fb L	75 fb 500
Atrazine	C	2997
Atrazine fb paraquat	C fb L	2997 fb 500
Simazine	C	1494
Simazine fb paraquat	C fb L	1494 fb 500
Terbuthylazine	C	750
Terbuthylazine fb paraquat	C fb L	750 fb 500
Haloxyfop	A	156
Haloxyfop fb paraquat	A fb L	156 fb 500
Paraquat	L	500
Paraquat fb paraquat	L fb L	500 fb 500

Abbreviations: fb = followed by 2nd application of herbicide 7 days later.

**Table 3:** Effect of single and tank mixture herbicide treatments on weed biomass at 42 d after treatment of flowering *Echinochloa colona* in pots over two separate experiments conducted in 2015 and 2016.

Treatments	Application Type	Dry Biomass (g plant^−1^)	
		2015	2016
Nontreated		7.72	8.12
Isoxaflutole	Single	2.25 (84)	5.75 (29)
Isoxaflutole plus paraquat	Tank mix	0.00 (100)	0.32 (96)
Atrazine	Single	3.47 (55)	3.55 (56)
Atrazine plus paraquat	Tank mix	0.00 (100)	0.67 (92)
Simazine	Single	5.92 (23)	6.47 (20)
Simazine plus paraquat	Tank mix	0.00 (100)	0.00 (100)
Terbuthylazine	Single	5.67 (27)	7.07 (13)
Terbuthylazine plus paraquat	Tank mix	0.00 (100)	0.05 (99)
Haloxyfop	Single	4.85 (37)	0.70 (91)
Haloxyfop plus paraquat	Tank mix	0.22 (97)	0.02 (99)
Paraquat	Single	0.38 (95)	0.23 (97)
LSD_0.05_		1.15	1.23

Values in parentheses are percentage control relative to nontreated. Abbreviations: LSD, least significant difference at the 5% level of significance (0.05).

**Table 4:** Effect of single and sequential herbicide treatments on weed biomass at 42 d after treatment of flowering *Echinochloa colona* in pots over two separate experiments conducted in 2015 and 2016.

Treatments	Application Type	Dry Biomass (g plant ^−1^)	
		2015	2016
Nontreated		8.68	9.52
Isoxaflutole	Single	3.50 (60)	5.10 (46)
Isoxaflutole fb paraquat	Sequential	0.02 (99)	0.00 (100)
Atrazine	Single	2.73 (69)	2.27 (76)
Atrazine fb paraquat	Sequential	0.03 (99)	0.00 (100)
Simazine	Single	6.90 (21)	4.47 (53)
Simazine fb paraquat	Sequential	0.10 (99)	0.00 (100)
Terbuthylazine	Single	5.23 (40)	5.18 (46)
Terbuthylazine fb paraquat	Sequential	0.32 (96)	0.02 (99)
Haloxyfop	Single	5.17 (40)	1.65 (83)
Haloxyfop fb paraquat	Sequential	0.00 (100)	0.00 (100)
Paraquat	Single	0.50 (94)	0.03 (99)
Paraquat fb paraquat	Sequential	0.00 (100)	0.00 (100)
LSD_0.05_		1.85	1.38

Values in parentheses are percentage control relative to nontreated. Abbreviations: fb = followed by 2nd application of herbicide 7 days later; LSD, least significant difference at the 5% level of significance (0.05).

**Table 5:** Effect of single and tank mixture herbicide treatments on weed biomass at 42 d after treatment of flowering *Chloris virgata* in pots over two separate experiments conducted in 2015 and 2016.

Treatments	Application Type	Dry Biomass (g plant^−1^)	
		2015	2016
Nontreated		12.80	15.62
Isoxaflutole	Single	3.45 (73)	4.93 (68)
Isoxaflutole plus paraquat	Tank mix	0.98 (92)	0.2 (99)
Atrazine	Single	10.30 (20)	8.65 (47)
Atrazine plus paraquat	Tank mix	1.60 (88)	0.72 (95)
Simazine	Single	6.13 (52)	9.63 (38)
Simazine plus paraquat	Tank mix	2.43 (96)	1.32 (92)
Terbuthylazine	Single	9.18 (28)	11.32 (28)
Terbuthylazine plus paraquat	Tank mix	1.65 (87)	0.50 (97)
Haloxyfop	Single	0.28 (98)	0.10 (99)
Haloxyfop plus paraquat	Tank mix	0.00 (100)	0.00 (100)
Paraquat	Single	3.62 (72)	2.03 (87)
LSD_0.05_		1.55	1.79

Values in parentheses are percentage control relative to nontreated. Abbreviations: LSD, least significant difference at the 5% level of significance (0.05).

**Table 6:** Effect of single and sequential herbicide treatments on weed biomass 42 d after treatment of flowering *Chloris virgata* in pots over two separate experiments conducted in 2015 and 2016.

Treatments	Application Type	Dry Biomass (g plant^−1^)	
		2015	2016
Nontreated		12.97	15.12
Isoxaflutole	Single	2.13 (84)	7.60 (50)
Isoxaflutole fb paraquat	Sequential	0.00 (100)	0.00 (100)
Atrazine	Single	8.95 (31)	7.05 (53)
Atrazine fb paraquat	Sequential	0.23 (98)	0.00 (100)
Simazine	Single	11.68 (10)	9.47 (37)
Simazine fb paraquat	Sequential	0.93 (93)	0.98 (94)
Terbuthylazine	Single	10.40 (20)	12.07 (20)
Terbuthylazine fb paraquat	Sequential	0.72 (94)	0.18 (99)
Haloxyfop	Single	0.55 (96)	0.32 (98)
Haloxyfop fb paraquat	Sequential	0.00 (100)	0.00 (100)
Paraquat	Single	2.32 (82)	1.40 (91)
Paraquat fb paraquat	Sequential	0.47 (96)	0.00 (100)
SD_0.05_		1.68	1.80

Values in parentheses are percentage control relative to nontreated. Abbreviations: fb = followed by 2nd application of herbicide 7 days later; LSD, least significant difference at the 5% level of significance (0.05).
